# Association of Dietary Components and Combined Dietary Profiles with Ulcerative Colitis: A Case–Control Study

**DOI:** 10.3390/diseases14080297

**Published:** 2026-08-18

**Authors:** Haytham A. Sheerah, Ahmed Arafa, Rola A. Jalloun, Mudi H. Alharbi, Haneen N. Molla, Amnah N. Yamani, Sahar A. Alqadi, Abdullah A. Asiri, Norah F. Saleh, Yazeed M. Alsaedi, Banan H. Mekwar, Anas Almofarreh

**Affiliations:** 1 International Collaboration Department at Chief Executive Office, Public Health Authority, Riyadh 13351, Saudi Arabia; 2 Population Health Deputyship, Ministry of Health, Riyadh 11176, Saudi Arabia; 3 Department of Public Health and Community Medicine, Faculty of Medicine, Beni-Suef University, Beni-Suef 62511, Egypt; 4 Clinical Nutrition Department, College of Applied Medical Sciences, Taibah University, Madinah 42353, Saudi Arabia; 5 Clinical Nutrition Department, King Khalid University Hospital, King Saud University Medical City, Riyadh 12372, Saudi Arabia; 6 Medical Laboratory Sciences Department, College of Applied Medical Sciences, King Abdulaziz University, Jeddah 21589, Saudi Arabia; 7 Global Health Division, Public Health Authority, Riyadh 13351, Saudi Arabia; 8 Aseer Cluster Health, Ministry of Health, Aseer 62523, Saudi Arabia; 9 Quality Outcomes Department, Ministry of Health, Aseer 62523, Saudi Arabia; 10 Department of Medicine, Alhada Armed Forces Hospital, Taif 21944, Saudi Arabia; 11 Deputyship for Therapeutic Services, Ministry of Health, Riyadh 11176, Saudi Arabia; 12 The Investment Deputyship, Ministry of Health, Riyadh 11176, Saudi Arabia

**Keywords:** intestinal inflammation, dietary patterns, fast food, dairy, carbonated soft drinks

## Abstract

**Background and Objectives**: Diet is a potential modifiable risk factor for ulcerative colitis (UC). This study examined the associations of individual dietary components and combined dietary profiles with UC. **Methods and Study Design**: This case–control study included 157 patients with UC and 395 controls. Dietary consumption frequencies were converted into ordinal scores ranging from 1 to 4. Participants were additionally classified into four dietary profiles based on combined levels of risk-promoting and protective food consumption. Logistic regression models were used to estimate odds ratios (ORs) and 95% confidence intervals (CIs). **Results**: Higher consumption of fast food (OR = 5.30, 95% CI: 3.65, 7.70), carbonated soft drinks (OR = 9.37, 95% CI: 5.78, 15.18), and spicy food (OR = 1.56, 95% CI: 1.21, 2.01) was positively associated with UC. Dairy consumption was inversely associated with UC (OR = 0.52, 95% CI: 0.33, 0.82). In sex-stratified analyses, vegetable consumption was inversely associated with UC among women only. Compared with participants with high risk-promoting/low protective food consumption, those with low risk-promoting/low protective food consumption (OR = 0.07, 95% CI: 0.02, 0.21) and those with low risk-promoting/high protective food consumption (OR = 0.08, 95% CI: 0.03, 0.17) had substantially lower odds of UC. The high risk-promoting/high protective food consumption was not associated with UC. **Conclusions:** Frequent consumption of fast food, carbonated soft drinks, and spicy food was associated with higher odds of UC, whereas dairy consumption was associated with lower odds. Lower consumption of risk-promoting foods, irrespective of protective food consumption, was associated with substantially lower odds of UC. Prospective studies are needed to confirm these associations.

## 1. Introduction

Ulcerative colitis (UC) is a chronic inflammatory disease of the colon characterized by recurrent episodes of mucosal inflammation and periods of remission and relapse. Patients commonly present with bloody diarrhea, rectal bleeding, abdominal pain, and urgency, and many require long-term medical therapy or surgery during the course of their disease [1,2]. Beyond its physical manifestations, UC is associated with impaired quality of life and an increased burden of anxiety, depression, and psychological distress [3]. According to a recent systematic review and meta-analysis of 215 population-based studies, the global prevalence and incidence of UC were estimated at 120.4 cases per 100,000 population and 5 cases per 100,000 person-years, respectively [4]. UC also imposes a substantial economic burden. Annual direct healthcare costs have been estimated at approximately US$7200 per case in Europe and US$13,600 per case in North America, with medication accounting for the largest proportion of healthcare expenditures [5]. Although UC has traditionally been more common in North America and Europe, its incidence and prevalence have increased steadily in newly industrialized regions, including the Middle East and North Africa (MENA) [6]. In the Arab world, the pooled annual incidence of UC is estimated at 2.3 cases per 100,000 population [7].

Diet is a plausible environmental determinant of UC. Evidence from experimental and epidemiological studies suggests that dietary habits influence disease susceptibility through effects on the gut microbiota, intestinal barrier integrity, and immune function [8,9,10,11,12]. In Arab countries, rapid urbanization has been accompanied by marked changes in dietary habits, characterized by increased consumption of processed foods, sugar-sweetened beverages, and other Western-style foods [13]. These dietary changes have occurred alongside the increasing incidence of UC in the region 7, raising the possibility that dietary factors contribute to disease risk.

In this context, we previously conducted a series of case–control studies using data from Saudi Arabia to examine the associations between individual dietary components and UC [14,15,16,17,18]. However, these studies evaluated each dietary component independently and did not account for the combined effects of multiple dietary exposures. They also relied on merged dietary consumption categories, limiting the assessment of dose–response associations, and did not evaluate overall dietary profiles. In addition, sex-specific associations were not examined consistently across dietary components despite evidence that dietary behaviors and immune responses differ between men and women [19,20]. Therefore, this study aimed to investigate the associations of individual dietary components and combined dietary profiles with UC using ordinal dietary consumption scores and dietary profiles reflecting joint exposure to risk-promoting and protective foods. Sex-stratified analyses were also performed to examine whether these associations differed between men and women.

## 2. Methods

### 2.1. Study Design and Population

This hospital-based case–control study used data collected between January 2009 and December 2017 at a private polyclinic in Riyadh, Saudi Arabia. Eligible cases were adults aged 18 years or older with newly diagnosed UC. The control group comprised adults attending the same clinic during the same period for gastrointestinal conditions other than inflammatory bowel disease (IBD), primarily gastroenteritis (87.1% of controls), with smaller numbers diagnosed with gastroesophageal reflux disease, irritable bowel syndrome, hemorrhoids, or gallstone disease. Controls had no history or clinical evidence of IBD, gastrointestinal malignancy, polyposis, or diverticulosis. Participants with missing information on the dietary variables included in the present analysis were excluded. The final analytical sample comprised 157 patients with UC and 395 controls.

### 2.2. Ulcerative Colitis Diagnosis

Patients presenting with symptoms suggestive of UC underwent clinical assessment and laboratory testing, including urine and stool analyses, complete blood count, and inflammatory markers. Patients with clinical and laboratory findings suggestive of UC subsequently underwent colonoscopy. Mucosal biopsy specimens obtained during colonoscopy were examined histopathologically, and only patients with colonoscopic and histological confirmation were included as cases.

### 2.3. Assessment of Dietary Consumption

Dietary intake was assessed using a self-administered questionnaire completed before the results of laboratory investigations, colonoscopy, and histopathological examination were available. Participants were asked to report their usual frequency of consuming fast food, carbonated soft drinks, spicy food, fruits, vegetables, dairy products, coffee, and tea during the period preceding the onset of their current gastrointestinal symptoms. Periods of fasting, including Ramadan, were excluded. For each food or beverage item, the response categories were “monthly or less,” “weekly,” “twice weekly,” and “daily.” The questionnaire measured consumption frequency but did not collect information on portion size, total energy intake, number of meals, or nutrient intake. Therefore, the dietary variables represent frequency of consumption rather than quantitative intake.

### 2.4. Covariates

Information on age, sex, and smoking behavior was obtained from the questionnaire. Weight and height were measured at the clinic, and body mass index (BMI) was calculated as weight in kilograms divided by height in meters squared (kg/m^2^). Laboratory measurements of inflammatory markers, including white blood cell count (WBC), erythrocyte sedimentation rate (ESR), and C-reactive protein (CRP), were obtained from medical records.

### 2.5. Statistical Analysis

Continuous variables are presented as means and standard deviations (SDs), and categorical variables as frequencies and percentages. Differences between patients with UC and controls were assessed using the independent-samples *t*-test for continuous variables and the chi-square test for categorical variables. Fisher’s exact test was used when expected cell frequencies were small. Dietary frequency categories were assigned ordinal scores of 1 to 4, corresponding to “monthly or less,” “weekly,” “twice weekly,” and “daily,” respectively. The resulting score for each dietary item was treated as an ordinal exposure, with the odds ratio representing the change in the odds of UC associated with a one-category increase in consumption frequency. Binary logistic regression was used to estimate odds ratios (ORs) and 95% confidence intervals (CIs) for the associations between dietary consumption scores and UC. Three models were fitted. Model I adjusted for age and sex. Model II additionally adjusted for BMI and smoking status. Model III further adjusted for WBC, ESR, and CRP. Sex-stratified models were fitted separately for men and women and adjusted for age, BMI, smoking status, and inflammatory markers.

A risk-promoting dietary score was calculated by averaging the ordinal scores for fast food, carbonated soft drinks, spicy food, and fruits. A protective dietary score was calculated by averaging the scores for vegetables, dairy products, coffee, and tea. For each dietary component, scores of 1 and 2 were classified as low consumption and scores of 3 and 4 as high consumption. The risk-promoting and protective classifications were then combined to form four dietary profiles: high risk-promoting/low protective, high risk-promoting/high protective, low risk-promoting/low protective, and low risk-promoting/high protective. The high risk-promoting/low protective profile was used as the reference category in logistic regression analyses. All analyses were performed using IBM SPSS Statistics for Windows, version 22.0. Statistical significance was defined as a two-sided *p*-value < 0.05.

## 3. Results

### 3.1. Participant Characteristics

The study included 157 patients with UC and 395 controls. Patients with UC were older than controls (39.9 ± 12.6 vs. 37.6 ± 8.9 years, *p* < 0.001) and had a lower BMI (25.1 ± 5.8 vs. 27.2 ± 6.4 kg/m^2^, *p* < 0.001). No significant differences were observed in sex distribution (*p* = 0.124) or smoking status (*p* = 0.058). Elevated WBC, ESR, and CRP were more common in patients with UC than controls (all *p* ≤ 0.001) (Table 1).

### 3.2. Dietary Consumption Frequency

**Figure 1:**
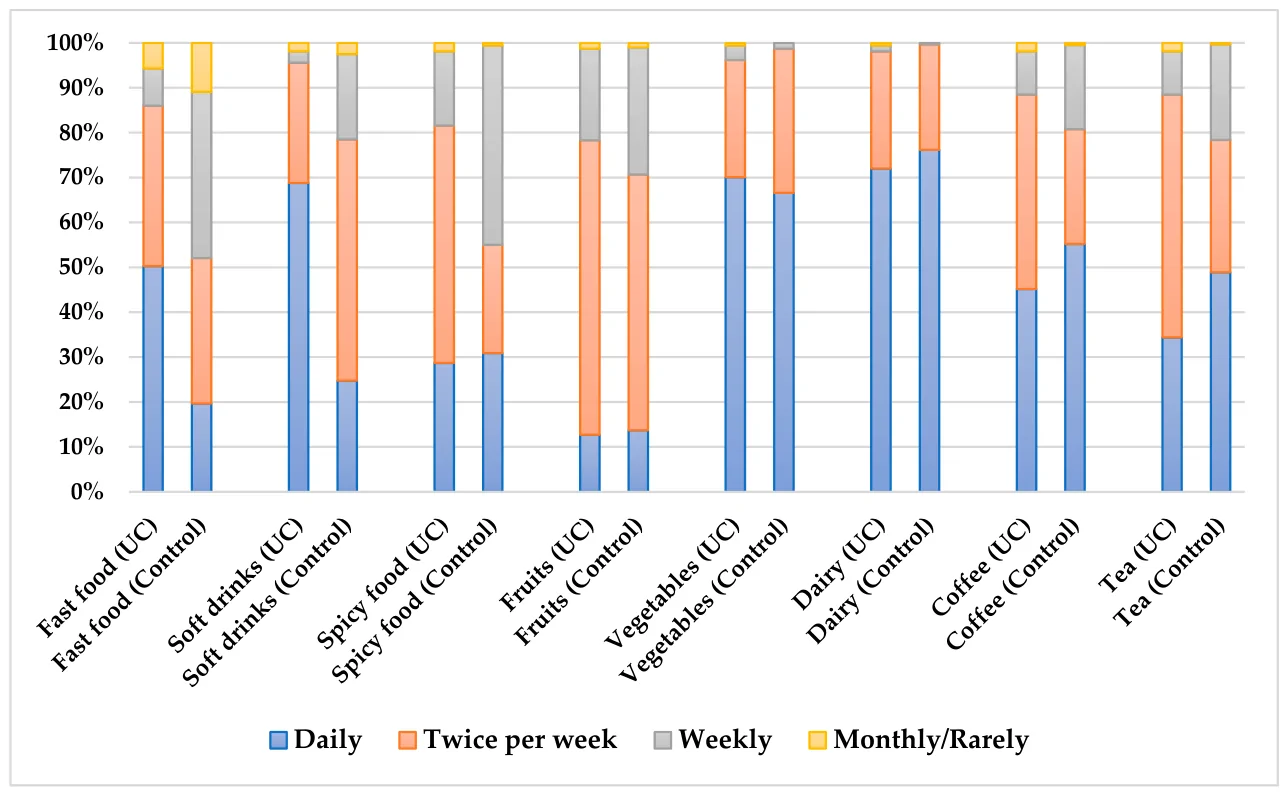
Distribution of dietary consumption frequencies among patients with ulcerative colitis and controls. Percentages indicate the proportion of participants in each dietary component.

Daily consumption of fast food and carbonated soft drinks was more frequent among patients with UC than controls (50.3% vs. 19.7% and 68.8% vs. 24.8%, respectively). Twice-weekly consumption of spicy food was also more frequent among patients with UC (52.9% vs. 24.1%). The distributions of fruit, vegetable, dairy product, coffee, and tea consumption were comparable between the two groups (Figure 1).

### 3.3. Dietary Consumption Scores

**Figure 2:**
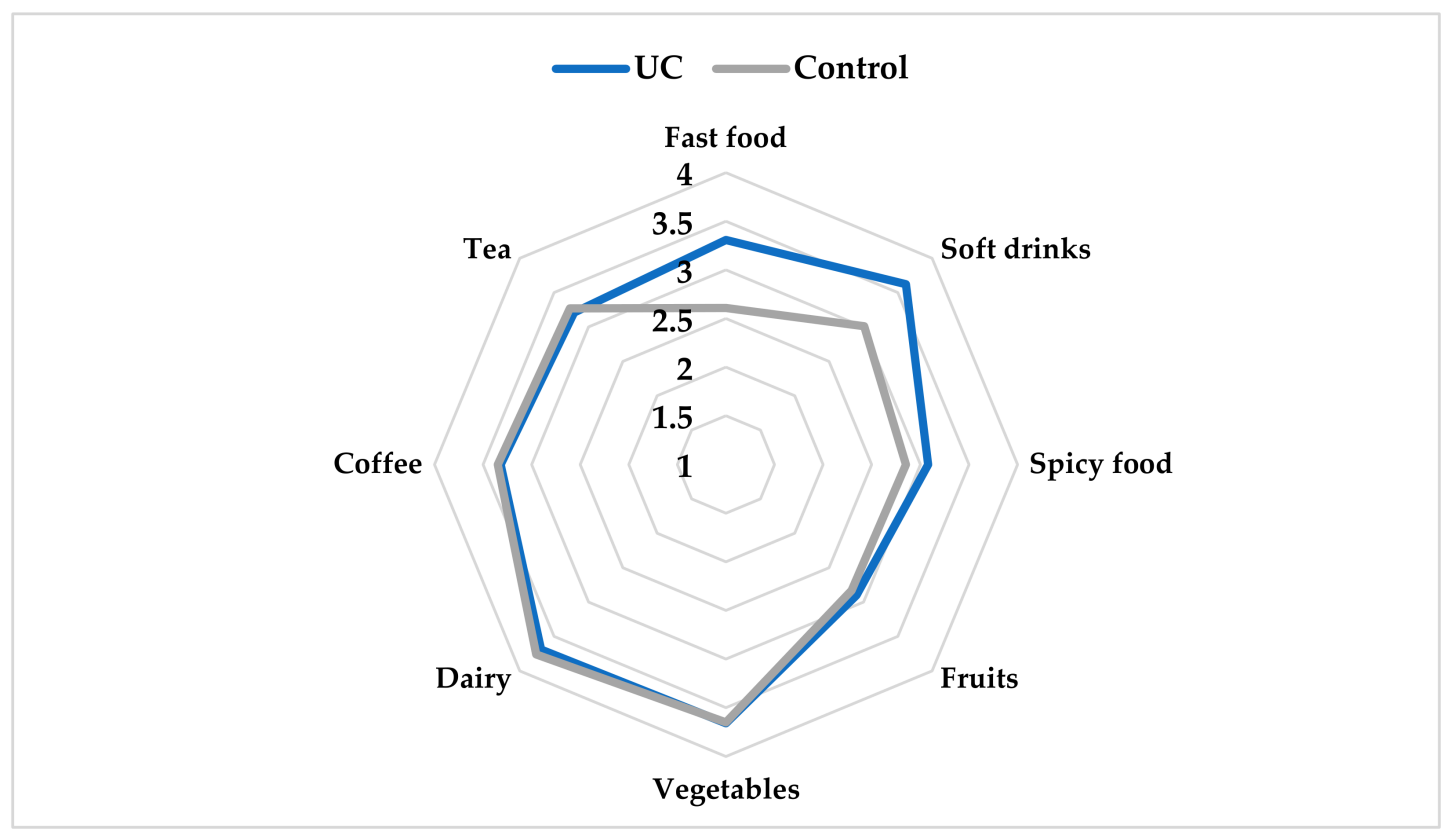
Mean dietary consumption scores among patients with ulcerative colitis and controls. Dietary consumption frequencies were converted into ordinal scores (1 = monthly or less, 2 = weekly, 3 = twice weekly, and 4 = daily).

Patients with UC had higher consumption scores for fast food (3.31 ± 0.85 vs. 2.61 ± 0.92; *p* < 0.001), carbonated soft drinks (3.62 ± 0.64 vs. 3.01 ± 0.74; *p* < 0.001), and spicy food (3.08 ± 0.73 vs. 2.85 ± 0.87; *p* = 0.003) than controls. No significant differences were observed for fruit, vegetables, dairy products, coffee, or tea (Table 2 and Figure 2).

### 3.4. Association Between Dietary Consumption Scores and Ulcerative Colitis

Higher consumption scores for fast food, carbonated soft drinks, and spicy food were positively associated with UC in all three regression models. The magnitude of these associations increased slightly after sequential adjustment for BMI, smoking status, and inflammatory markers. In the fully adjusted model (Model III), each one-category increase in the consumption score was associated with higher odds of UC for fast food (OR = 5.30, 95% CI: 3.65, 7.70), carbonated soft drinks (OR = 9.37, 95% CI: 5.78, 15.18), and spicy food (OR = 1.56, 95% CI: 1.21, 2.01). Dairy consumption was inversely associated with UC across all three models, with the association strengthening after adjustment (Model III: OR = 0.52, 95% CI: 0.33, 0.82). No statistically significant associations were observed for fruit, vegetables, coffee, or tea in any of the regression models (Table 3).

### 3.5. Sex-Stratified Analyses

Higher fast food and carbonated soft drink consumption were associated with increased odds of UC in both men (OR = 5.21, 95% CI: 3.12, 8.70 and OR = 9.86, 95% CI: 5.00, 19.45, respectively) and women (OR = 5.54, 95% CI: 3.07, 10.02 and OR = 10.74, 95% CI: 4.91, 23.48, respectively). Spicy food consumption was positively associated with UC among men (OR = 1.91, 95% CI: 1.34, 2.72) but not women (OR = 1.25, 95% CI: 0.84, 1.85). Vegetable consumption was inversely associated with UC among women (OR = 0.40, 95% CI: 0.18, 0.87), whereas dairy consumption was inversely associated with UC among men (OR = 0.43, 95% CI: 0.24, 0.78). No significant associations were observed for fruit, coffee, or tea in either sex (Table 4).

### 3.6. Dietary Profiles

**Figure 3:**
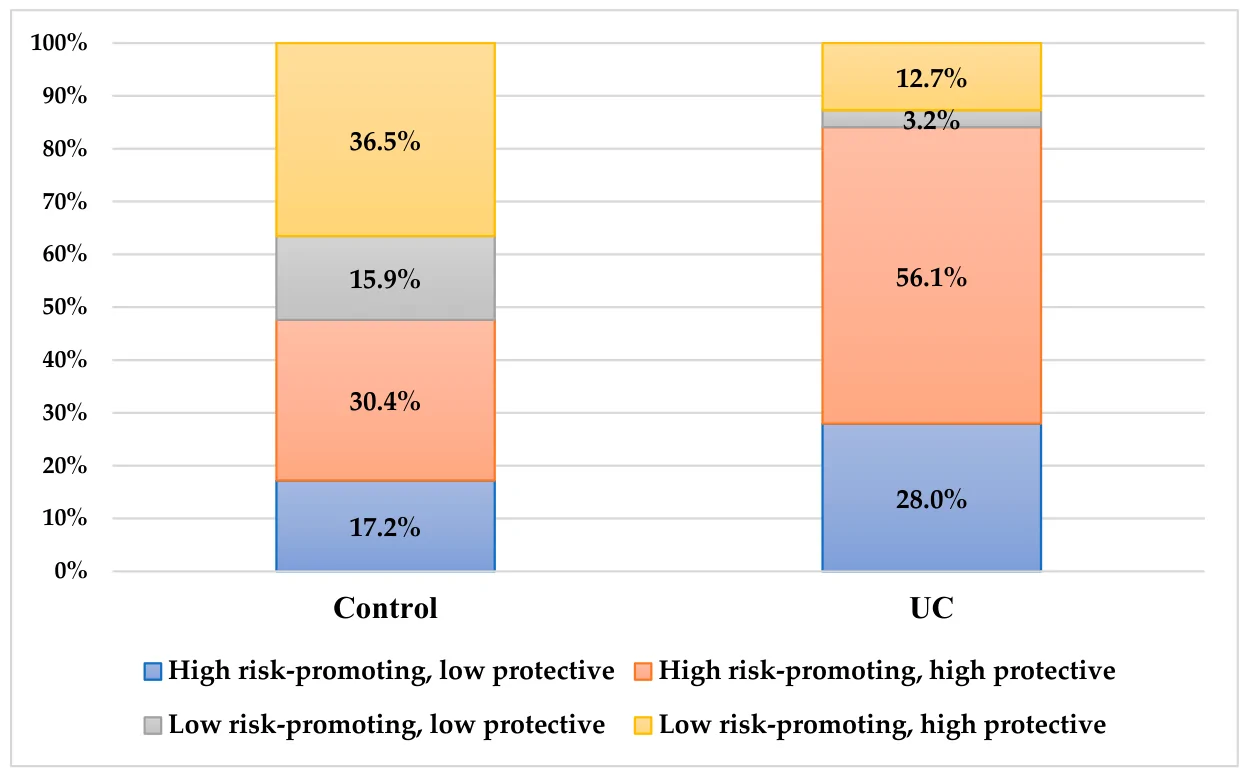
Distribution of combined dietary profiles among patients with ulcerative colitis and controls. Dietary profiles were defined according to combined levels of risk-promoting and protective food consumption: high risk-promoting/low protective, high risk-promoting/high protective, low risk-promoting/low protective, and low risk-promoting/high protective. Percentages indicate the proportion of participants in each dietary profile.

The low risk-promoting/high protective profile was observed in 36.5% of controls and 12.7% of patients with UC. The high risk-promoting/high protective profile was observed in 30.4% of controls and 56.1% of patients with UC. The high risk-promoting/low protective profile accounted for 17.2% of controls and 28.0% of patients with UC, whereas the low risk-promoting/low protective profile accounted for 15.9% and 3.2%, respectively (Figure 3).

Compared with the high risk-promoting/low protective profile, the low risk-promoting/low protective profile (OR = 0.07, 95% CI: 0.02, 0.21) and the low risk-promoting/high protective profile (OR = 0.08, 95% CI: 0.03, 0.17) were associated with lower odds of UC in the fully adjusted model. The high risk-promoting/high protective profile was not significantly associated with UC (Table 5).

## 4. Discussion

This study evaluated the associations of individual dietary components and overall dietary profiles with UC in an Arab population. Higher consumption of fast food, carbonated soft drinks, and spicy food was associated with higher odds of UC. These findings are consistent with our previous studies using the same source population [14,17,18]. However, those studies evaluated each dietary component separately after merging the original consumption categories into broader exposure groups. In the present study, the original ordinal consumption categories were retained. The present findings are also consistent with previous epidemiological evidence. In a study of Romanian and Belgian populations, Preda et al. found that patients with IBD had higher consumption of sweets and sweetened beverages (OR = 3.36; 95% CI: 1.60, 7.00), processed and high-fat meat (OR = 2.50; 95% CI: 1.40, 4.70), and fried foods (OR = 9.50; 95% CI: 3.80, 23.60) than healthy controls [21]. In a subsequent study that examined IBD subtypes separately, patients with UC reported more frequent consumption of sweetened beverages (47.5% vs. 17.2%, *p* = 0.005), fatty meat (55.0% vs. 28.1%, *p* = 0.025), and fried foods (55.0% vs. 9.4%, *p* < 0.001) than healthy controls [22]. A meta-analysis of nine studies reported that adherence to a Western dietary pattern was associated with higher UC risk (RR = 2.15; 95% CI: 1.38, 3.34) [23]. A meta-analysis of five studies showed that higher soft drink consumption was associated with increased UC risk (RR = 1.69; 95% CI: 1.24, 2.30) [24]. More recent studies have also reported positive associations between dietary patterns characterized by processed foods, refined carbohydrates, and sugar-sweetened beverages and UC [10,11]. On the other hand, spices were shown to worsen IBD symptoms [25]. 

Several biological mechanisms may explain these associations. Fast foods are typically rich in saturated fat, refined carbohydrates, salt, food additives, and emulsifiers while providing relatively small amounts of dietary fiber. Experimental studies suggest that Western-style diets rich in these components alter gut microbial composition, impair epithelial barrier integrity, reduce short-chain fatty acid production, and activate pro-inflammatory immune pathways [12,23,26,27]. Carbonated soft drinks contribute substantially to dietary intake of added sugars, particularly fructose-containing sweeteners, which have been associated with gut microbial dysbiosis, increased intestinal permeability, and chronic low-grade inflammation [28]. Capsaicin, the principal bioactive compound in chili peppers, has also been shown experimentally to influence intestinal permeability, visceral sensitivity, gut microbial composition, and neurogenic inflammatory pathways through activation of transient receptor potential vanilloid-1 (TRPV1) receptors [29,30].

Higher dairy consumption was associated with lower odds of UC, whereas fruit, vegetables, coffee, and tea were not associated with UC in the overall analysis. The inverse association with dairy is consistent with our previous case–control study using the same source population and with a subsequent meta-analysis that reported lower odds of UC among individuals with the highest dairy consumption (pooled OR = 0.82; 95% CI: 0.68, 0.98), with similar results after exclusion of our study [15]. Dairy products provide calcium, vitamin D, high-quality proteins, bioactive peptides, and, in fermented products, probiotic microorganisms, all of which have been suggested to support epithelial barrier function, modulate immune responses, and reduce intestinal inflammation [31,32]. The inverse association between dairy consumption and UC was observed among men but not women. This finding should be interpreted with caution because relatively few women were classified in the monthly and weekly dairy consumption categories, which may have limited the statistical power of the sex-stratified analysis. Differences in dietary habits, including dairy consumption [33], and sex-specific differences in immune responses and the pathogenesis of IBD [19,20] may also have contributed to the observed findings.

Our study also showed an inverse association between vegetable consumption and UC among women. A meta-analysis of four prospective studies reported that higher vegetable consumption was associated with lower UC risk (RR = 0.56; 95% CI: 0.48, 0.66) [34]. Vegetables are major sources of dietary fiber, vitamins, minerals, and polyphenols that contribute to gut microbial diversity, short-chain fatty acid production, maintenance of epithelial barrier integrity, and regulation of mucosal immune responses [35]. Although women generally consume more vegetables than men [36], previous epidemiological studies have not reported sex-stratified analyses. Therefore, it remains unclear whether the observed association reflects true sex-specific differences or variation in dietary habits between men and women. In contrast, our previous study found inverse associations between frequent coffee and tea consumption and UC [14]. Those findings were based on merged dietary consumption categories, whereas the present study retained the original ordinal categories to evaluate dose–response relationships. No significant dose–response associations were observed for either coffee or tea.

An additional contribution of the present study was the evaluation of combined food consumption profiles. Most previous studies have examined individual foods or nutrients, whereas relatively few have evaluated combinations of dietary components. A recent case–control study used the Dietary Inflammatory Index (DII), which combines multiple dietary components into a single score reflecting the overall inflammatory potential of the diet, and found that participants with the highest DII scores had significantly higher odds of UC than those with the lowest scores (adjusted OR = 5.18; 95% CI: 1.43, 18.80). Furthermore, each one-unit increase in the DII score was associated with a 31% increase in the odds of UC (adjusted OR = 1.31; 95% CI: 1.09, 1.57) [37]. Another case–control study identified dietary patterns using factor analysis and found that adherence to a healthy dietary pattern was associated with a 79% lower odds of UC (OR = 0.21; 95% CI: 0.07, 0.59), whereas adherence to an unhealthy dietary pattern was associated with higher odds of UC (OR = 3.39; 95% CI: 1.16, 9.90) [38]. An earlier case–control study evaluated the combined consumption of Western foods, including bread, butter, margarine, cheese, meat, ham, and sausage, and reported that greater consumption of this dietary pattern was associated with an increased risk of UC (trend, *p* = 0.04) [39]. The present study differs from these studies by classifying participants according to both risk-promoting and protective food consumption. Participants with lower consumption of risk-promoting foods had lower odds of UC regardless of their consumption of protective foods, whereas higher consumption of protective foods alone was not associated with similarly lower odds among participants who frequently consumed risk-promoting foods. These findings suggest that the lower odds of UC observed across dietary profiles were more strongly related to lower consumption of risk-promoting foods than to higher consumption of protective foods.

This study has several strengths. First, we analyzed multiple dietary components at the same time instead of examining each food separately. Second, we used ordinal consumption scores to evaluate dose–response relationships. Third, we evaluated combined dietary profiles based on both risk-promoting and protective food consumption. Fourth, diagnoses of UC were confirmed using colonoscopy and histopathological examination.

However, several limitations should be considered. First, the case–control design precludes establishing temporality. Although participants were asked to report their usual dietary consumption before the onset of symptoms, some patients may have modified their diet in response to early or undiagnosed gastrointestinal symptoms. Such changes could result in reverse causation and may be particularly relevant to foods commonly avoided or increased during gastrointestinal symptoms, such as spicy foods. Second, dietary information was self-reported retrospectively and is therefore subject to recall and reporting bias. The dietary questionnaire assessed frequency of consumption but did not collect information on portion size, total energy intake, number of meals, specific food products, preparation methods, or nutrient intake. Consequently, participants with the same consumption frequency may have differed considerably in the actual amounts consumed, and the observed associations cannot be separated from differences in total energy intake or other aspects of diet. Third, the four frequency categories provide a relatively simple measure of habitual consumption and may not fully capture long-term dietary patterns. The questionnaire also covered a limited number of food groups and therefore did not provide a comprehensive assessment of the participants’ entire diet. Fourth, the controls were patients attending the same clinic for other gastrointestinal conditions rather than healthy population controls. Some of these conditions, particularly gastroenteritis and irritable bowel syndrome, may themselves be associated with dietary changes. This could have affected the reported consumption of some foods and may limit comparisons with the general population. Fifth, although the regression models accounted for age, sex, BMI, smoking, and inflammatory markers, residual confounding by unmeasured factors remains possible. These include socioeconomic status, physical activity, medication use, family history of IBD, antibiotic exposure, and other dietary factors not included in the questionnaire. Sixth, some sex-stratified analyses included relatively small numbers of participants within individual consumption categories. This was particularly evident for women in some dairy consumption categories and may have reduced the precision of the sex-specific estimates. The observed differences between men and women should therefore be interpreted cautiously. Seventh, the dietary profile and food protection score were constructed from the dietary variables available in this dataset and have not been externally validated. The classification of foods as risk-promoting or protective was based on previous evidence from the same population, and the resulting profiles should be evaluated in independent populations. Eighth, participants were recruited from a single private polyclinic in Riyadh between 2009 and 2017. Dietary habits in Saudi Arabia have continued to change since that period, and patients attending a private gastrointestinal clinic may differ from the wider Saudi or Arab population.

## 5. Conclusions

Higher consumption of fast food, carbonated soft drinks, and spicy food was associated with higher odds of UC, whereas dairy consumption was inversely associated with UC. When dietary components were considered together, lower consumption of risk-promoting foods was associated with lower odds of UC regardless of the level of protective food consumption. In contrast, higher consumption of protective foods did not appear to offset the association with frequent consumption of risk-promoting foods. Prospective studies using comprehensive dietary assessment are needed to confirm these findings and establish their temporal relationship with UC.

## Figures and Tables

**Table 1:** Comparison of sociodemographic and clinical characteristics between ulcerative colitis and control groups.

Characteristics		Ulcerative Colitis n = 157	Control n = 395	*p*-Value
Age (years), mean ± SD		39.9 ± 12.6	37.6 ± 8.9	<0.001
Sex, %	Men	59.2	64.8	0.124
	Women	40.8	35.2	
Body mass index (kg/m^2^), mean ± SD		25.1 ± 5.8	27.2 ± 6.4	<0.001
Smoking, %	No	88.6	79.3	0.058
	<10 cig/day	1.9	1.0	
	10–20 cig/day	7.0	12.9	
	>20 cig/day	2.5	6.8	
Elevated WBC, %		8.7	1.7	<0.001
Elevated ESR, %		47.8	20.6	<0.001
Elevated CRP, %		35.2	13.6	<0.001

WBC: White blood cell count, ESR: Erythrocyte sedimentation rate, CRP: C-reactive protein, SD: Standard deviation.

**Table 2:** Comparison of average dietary consumption scores between ulcerative colitis and control groups.

Food Items	Ulcerative Colitis Mean ± SD	Control Mean ± SD	*p*-Value
Fast food	3.31 ± 0.85	2.61 ± 0.92	<0.001
Soft drinks	3.62 ± 0.64	3.01 ± 0.74	<0.001
Spicy food	3.08 ± 0.73	2.85 ± 0.87	0.003
Fruits	2.90 ± 0.61	2.83 ± 0.66	0.254
Vegetables	3.66 ± 0.57	3.65 ± 0.50	0.955
Dairy	3.69 ± 0.53	3.76 ± 0.43	0.163
Coffee	3.32 ± 0.73	3.35 ± 0.80	0.633
Tea	3.21 ± 0.69	3.27 ± 0.80	0.414

SD: Standard deviation.

**Table 3:** Logistic regression of the association between dietary consumption scores and ulcerative colitis.

Food Items	Ulcerative Colitis		
	Model I OR (95% CI)	Model II OR (95% CI)	Model III OR (95% CI)
Fast food	4.14 (3.03, 5.66)	4.56 (3.27, 6.38)	5.30 (3.65, 7.70)
Soft drinks	6.46 (4.31, 9.67)	7.58 (4.92, 11.68)	9.37 (5.78, 15.18)
Spicy food	1.41 (1.12, 1.77)	1.48 (1.17, 1.88)	1.56 (1.21, 2.01)
Fruits	1.03 (0.75, 1.40)	1.04 (0.75, 1.44)	1.11 (0.79, 1.58)
Vegetables	0.82 (0.56, 1.21)	0.83 (0.56, 1.24)	0.82 (0.53, 1.25)
Dairy	0.61 (0.41, 0.92)	0.57 (0.37, 0.87)	0.52 (0.33, 0.82)
Coffee	0.92 (0.72, 1.17)	0.98 (0.77, 1.26)	1.04 (0.80, 1.36)
Tea	0.90 (0.70, 1.15)	0.96 (0.74, 1.24)	0.96 (0.73, 1.26)

OR: Odds ratio; Model I: ORs adjusted for age and sex; Model II: further adjusted for BMI and smoking; Model III: further adjusted for inflammatory markers.

**Table 4:** Logistic regression of the association between dietary consumption scores and ulcerative colitis in men and women.

Food Items	Ulcerative Colitis	
	Men OR (95% CI)	Women OR (95% CI)
Fast food	5.21 (3.12, 8.70)	5.54 (3.07, 10.02)
Soft drinks	9.86 (5.00, 19.45)	10.74 (4.91, 23.48)
Spicy food	1.91 (1.34, 2.72)	1.25 (0.84, 1.85)
Fruits	1.19 (0.75, 1.89)	1.04 (0.57, 1.87)
Vegetables	1.13 (0.64, 1.99)	0.40 (0.18, 0.87)
Dairy	0.43 (0.24, 0.78)	0.65 (0.30, 1.41)
Coffee	1.06 (0.73, 1.54)	1.06 (0.70, 1.60)
Tea	1.19 (0.82, 1.73)	0.81 (0.52, 1.25)

OR: Odds ratio; ORs adjusted for age, BMI, smoking, and inflammatory markers.

**Table 5:** Logistic regression of the association between dietary profiles and ulcerative colitis.

Dietary Profiles	Ulcerative Colitis		
	Model I OR (95% CI)	Model II OR (95% CI)	Model III OR (95% CI)
High risk-promoting/low protective	Ref	Ref	Ref
High risk-promoting/high protective	0.81 (0.49, 1.33)	0.89 (0.53, 1.50)	0.89 (0.51, 1.56)
Low risk-promoting/low protective	0.08 (0.03, 0.23)	0.07 (0.03, 0.20)	0.07 (0.02, 0.21)
Low risk-promoting/high protective	0.10 (0.05, 0.20)	0.10 (0.05, 0.20)	0.08 (0.03, 0.17)

OR: Odds ratio; Model I: ORs adjusted for age and sex; Model II: further adjusted for BMI and smoking; Model III: further adjusted for inflammatory markers.

## Data Availability

We did not make our data publicly available due to ethical or administrative reasons.
